# *E*x *vivo* cytokine production in psoriatic disease: Towards specific signatures in cutaneous psoriasis and peripheral psoriatic arthritis

**DOI:** 10.3389/fimmu.2022.993363

**Published:** 2022-11-08

**Authors:** Guillaume Larid, Adriana Delwail, Thomas Dalle, Philippe Vasseur, Christine Silvain, Jean-François Jégou, Franck Morel, Jean-Claude Lecron, Elisabeth Gervais

**Affiliations:** ^1^ Rheumatology Department, CHU de Poitiers, Poitiers, France; ^2^ University of Poitiers, LITEC, Poitiers, France; ^3^ UMR 6041, 4CS, Université de Poitiers, Poitiers, France; ^4^ Gastroenterology Department, CHU de Poitiers, Poitiers, France; ^5^ Immunology-Inflammation Laboratory, CHU de Poitiers, Poitiers, France

**Keywords:** psoriasis, psoriatic arthritis (PsA), psoriatic disease, cytokine signature, *ex vivo* stimulation

## Abstract

**Objectives:**

Psoriatic arthritis (PsA) and cutaneous psoriasis (PsO) are different phenotypes of psoriatic disease (PsD), whose underlying specific mechanisms remain incompletely understood. As cytokines are key elements to induce and tune up immune responses to drive inflammatory diseases, our objective was to assess whether clinical features, disease phenotype and PsA and PsO activity were associated with a particular *ex vivo* cytokine production profile.

**Methods:**

Forty-eight patients (37 PsA and 11 PsO) and 11 healthy subjects (HS) were studied. Cytokine production by peripheral blood mononuclear cells (PBMC) that were either unstimulated, or stimulated with LPS or anti-CD3/CD28 antibodies, were analysed by multiplex assay in the culture supernatants.

**Results:**

Cytokine signature of PsD includes a high level of TNFα in supernatants of LPS-stimulated PBMC, higher levels of IL-6 and lower levels of IFN-γ and IL-17A after CD3-CD28 stimulation, as well as higher spontaneous IL-1RA and TNFα production compared to HS. High body mass index (BMI) was associated with lower levels of IL-1β, and metabolic syndrome with lower levels of IFN-γ after LPS stimulation. In PsD, dermatological activity was related with higher IL-17A level, while rheumatic activity was linked with lower levels of IFN-γ and TNFα. Comparing each PsD subtype to HS, IL-1β and IL-6 productions are higher when using LPS stimulation in PsO patients with higher levels of IL-1β and IL-1α in peripheral PsA patients after CD3/CD28 stimulation. LPS stimulation induced high levels of IL-17A in peripheral PsA compared to axial PsA. PsA patients with axial PsA share some features with PsO but shows a distinct cytokine pattern compared to peripheral PsA.

**Conclusion:**

PsO and the different PsA subtypes exhibit distinct *ex vivo* cytokine production profiles and common features of the so-called PsD. Analysis of IL-1 cytokine family and IL-6 seems to be of particular interest to distinguish PsO and peripheral PsA since it depends on monocytes in PsO and T-lymphocytes in peripheral PsA. Peripheral cytokine profiles are influenced by rheumatic and dermatological activity of the disease, and also by metabolic syndrome features. Our results highlight the crucial role of immune cell interactions with different patterns of interaction depending on clinical phenotype.

## 1 Introduction

Psoriasis is an inflammatory cutaneous disorder with prevalence up to 3% of people in industrialized countries (1). Originally described as a skin and/or articular pathology, psoriasis appears as a disease involving other tissue damage. In the last ten years, the concept of “psoriatic disease” (PsD) has emerged, which encompasses all the clinical aspects of so-called cutaneous psoriasis (PsO), psoriatic arthritis (PsA), and associated comorbidities such as cardio-metabolic disorders (2–5). As PsD is a systemic disease, patients can present features of PsO, PsA, or both. Numerous studies focusing on PsO patients do not indicate if patients have articular involvement or not. Likewise, among PsO patients, many patients suffering from PsA are undiagnosed (6). This common vagueness in population’s description creates a potential bias both for the clinical diagnosis which determines the follow-up and treatment of patients, and for studies describing the physiopathological mechanisms of PsD, in which the two clinical entities may not be clearly differentiated from one to another.

PsD is a polygenic inflammatory disorder of which the pathophysiology raises numerous questions (7, 8). Interestingly, among the multiple polymorphisms associated with PsO or PsA, a significant number involves genes encoding for cytokines such as *IL-12B*, *IL-23A*, *IL-23R*, *IL-13*, *IL-36G, IL-20, TNFAIP3* or *TNFIP1* (7–9), leading to investigations of cytokine profiles in PsO or PsA in order to determine specific cytokine signatures. To date, different biological approaches have been applied to PsD, including serum analysis, transcriptomic analysis, and *ex vivo* studies, but the majority of these studies do not discriminate between the two clinical entities. While transcriptomic approaches from inflamed skin or synovial tissue samples provide relevant information on the pathophysiology of both diseases, their use in routine practice is not possible for diagnosis, insofar it requires invasive techniques to obtain samples. Therefore, most studies have focused on cytokine assays from patient blood samples, even though it is known that circulating cytokines are the tip of the iceberg for tissue inflammatory diseases. The conflicting data between studies and/or between subjects have confirmed the fact that measurement of serum cytokines such as TNF-α, IL-1α, IL-1β, IL-17, IL-23, IL-18 or IL-33 is not a reliable and reproducible method to determine cytokine status in PsD (10–15).

An alternative approach would consist in analysing the cytokine secretion capacities of circulating immune cells so as to segregate between PsA and PsO. Studies investigating cytokine secretion profiles from *ex vivo*-stimulated PBMC have been reported in PsO and PsA patients. Nishibu et al. studied spontaneous secretions of a limited number of cytokines (IL-1β, IL-2, IL-6, IL-8 and IFN-γ) and reported higher secretions of IL-1β, IL-6, and IL-8 by peripheral blood mononuclear cells (PBMC) isolated from PsA patients compared to PsO patients and from PsO patients compared to healthy subjects (16). However, this cytokine panel did not include relevant cytokines such as TNF-α or IL-17A. In a second study by Bosè et al., the authors also used a limited panel of T cell cytokines composed of IL-2, IFN-γ, TNF-α, IL-4, IL-5, IL-10, and IL-17 and showed that higher IFN-γ and IL-10 levels were produced by activated PBMC from PsO compared to healthy subjects (17). Finally, cytokine secretion was induced after stimulation using an anti-CD3 monoclonal antibody (mAb). This approach does not take into account non-T cell populations as potential sources of cytokines, and hides possible interactions between activated non-T cells and T cells.

The objective of the present study was to assess the cytokine profiles of PsA and PsO patients by different *ex vivo* PBMC stimulations in order to highlight potential differences between these two distinct pathological entities. Correlation with the clinical variables of metabolic syndrome (MetS), dermatological activity, and rheumatic activity will likewise be explored, as will the differences before and after DMARD (disease modifying anti-rheumatic drugs) treatment initiation in PsA patients.

## 2 Materials and methods

### 2.1 Patients

Patients with PsA were included if they were 18-years-old or older and fulfilled the Classification Criteria for Psoriatic Arthritis (CASPAR) of PsA (18). Patients of 18-years-old or older with PsO diagnosed by dermatologist without any joint complaint were included. Healthy controls were included if they did not suffer from any rheumatic, dermatological or inflammatory condition.

Patients were followed in outpatient clinics and included at any time point of the disease.

Characteristics of patients including dermatological activity, rheumatic activity, CRP sera levels, BMI, HOMA score, PASI, psoriasis duration, age, and abdominal circumference were obtained in the patients’ medical records. Detailed clinical characteristics were available for 31 out of the 37 PsA patients, who were classified as having axial disease, peripheral disease, or both patterns (mixed).

Dermatological activity (i.e. moderate to severe disease) was defined as PASI > 10 (19) and rheumatic activity as DAS28 ≥ 2.6 for peripheral PsA (20), BASDAI ≥ 40/100 for axial PsA (21), and either one of those two criteria for mixed PsA. Overall clinical activity was defined as an active disease that was either dermatological, or rheumatic, or both.

Focusing on metabolic syndrome (MetS) features, high abdominal circumference was defined as above 88 cm for women and 102 cm for men. HOMA score was considered elevated when superior to 3.

High CRP was defined as CRP ≥ 3 mg/L, and MetS was defined according to the ATP III definition (22).

We separately analysed 6 patients for whom we had samples taken before and after DMARD initiation, either csDMARD (methotrexate for 1) or bDMARD (TNF-α inhibitors for 5 of them).

### 2.2 Samples and cell culture

Ten millilitres of blood were collected from patients and healthy controls in Vacutainer tubes containing heparin for the isolation of PBMCs. Human PBMCs were isolated by density gradient centrifugation using Ficoll-Paque (GE Healthcare) and counted. Analysis of cell viability has been performed using Trypan Blue 0.4% (Merck-Sigma). All samples displayed at least 95% of cell viability. Isolated PBMCs were cultured in RPMI 1640 containing 2 mM glutamine, 100 U/ml penicillin-streptomycin (Invitrogen Life Technologies), and 10% heat-inactivated foetal calf serum (Gibco Life Technologies). Cells were seeded at 1 × 10^6^ cells/ml in 24-well plates and incubated 24 h at 37°C in a humidified atmosphere containing 5% CO_2_. Cultures were either left unstimulated to reflect the intrinsic state of cell activation or stimulated throughout the 24 h culture period with *E. coli* LPS (1 µg/ml; Sigma L2654 – *Escherichia Coli* serotype 026:B6) for monocyte activation or anti-CD3/CD28 mAb coated-beads for T cell activation (2.5 × 10^5^ beads/10^6^ cells/ml; Invitrogen Life Technologies) (23, 24). After incubation, the plates were centrifuged and supernatants were stored at -80°C until analysis. To estimate lymphocyte activity, the secretion of pro- and anti-inflammatory cytokines has been measured with a set of cytokines representative of Th subsets. Even if they were not exclusively secreted by these Th subsets, IFN-γ, IL-4, IL-17, and IL-10 were representative of Th1 cell, Th2 cell, Th17 cell, and Treg cell, respectively (25).

### 2.3 Cytokine measurements

The concentrations of IL-1α, IL-1β, IL-1RA, IL-4, IL-17, IL-6, TNF-α, IL-10, and γ interferon (IFN-γ) were quantified in cell culture supernatants using the MILLIPLEX MAP Human Cytokine/Chemokine magnetic bead panel kit (Millipore Corporation, Billerica, MA) according to the manufacturer’s instructions. Data were obtained with the Luminex 200™ platform (Luminex Xmap Technology) and analysed with the xPONENT™ software. All samples were assayed in duplicates.

### 2.4 Ethics

All patients gave informed consent. The study was conducted in accordance with the declaration of HELSINKI and was approved by the Poitiers University Hospital research board and local ethics committee.

### 2.5 Statistical analysis

All statistical analyses were performed using GraphPad Prism 9 (GraphPad Software, Inc.). Quantitative data were described by means and standard deviation or median. Qualitative data were presented as absolute frequencies and percentages.

The nonparametric Mann-Whitney U test was used to evaluate the difference of quantitative variables between two groups. For comparison of quantitative variables between three groups, Kruskal-Wallis test with *post-hoc* Dunn’s multiple comparison tests were performed. Paired observations were compared with the Wilcoxon matched-pairs test. Chi-square tests or Fisher’s exact test were conducted to compare qualitative variables between groups. All box plots showed medians with the minimal and maximal values. *P* values less than 0.05 were considered statistically significant.

## 3 Results

### 3.1 Description of the patient population

Thirty-seven PsA patients and 11 patients with PsO were included in the study, as were 11 healthy subjects. The population had a mean age of 53 years and 62.5% were women. MetS was present in 33.3% of PsD patients, with a mean BMI of 28 kg/m², a mean HOMA score of 3.74, and a mean abdominal circumference of 96.4 cm for women and 101.5 cm for men. In PsA patients, 37.8% had a purely peripheral form of the disease, 16.2% a purely axial disease, and 46% a mixed pattern. In PsO and PsA respectively, 63.6% and 67.6% of patients received csDMARDs, while 54.5% and 40.5% received bDMARDs. The mean PASI score was higher in PsO (7.42) than in PsA patients (3.69) (p = 0.030), with 27.2% and 12.9% respectively having a dermatologically active disease. There were no other significant differences between PsO, PsA and PsD groups (**Table 1**).

**Table 1:** Description of the population.

		PsO (n = 11)	PsA (n = 37)	PsD (n = 48)	p	p ; PsA vs PsO	p ; PsA vs PsD	p ; PsO vs PsD
Age [mean (SD); years]		57.82 (12.41)	51 (13.17)	53 (13.19)	0.215	0.239	> 0.999	0.495
Female patients (n; %)		9 (81.8%)	21 (56.8%)	30 (62.5%)	0.321	0.171	0.592	0.302
PsA Phenotype (n; %)								
Axial PsA			6 (16.2%)		–	–	–	–
Peripheral PsA			14 (37.8%)		–	–	–	–
Mixed PsA			17 (46%)		–	–	–	–
Treatment								
csDMARD (n; %)		7 (63.6%)	25 (67.6%)	32 (66.7%)	0.971	0.843	0.930	0.848
bDMARD (n; %)		6 (54.5%)	15 (40.5%)	21 (43.8%)	0.713	0.498	0.767	0.517
TNF inhibitors (n; %)		4 (66.7%)	13 (86.7%)	17 (81.0%)	0.997	> 0.999	0.979	> 0.999
Ustekinumab (n; %)		2 (33.3%)	1 (6.7%)	3 (14.3%)	NR	NR	NR	NR
Rituximab (n; %)		0 (0%)	1 (6.7%)	1 (4.8%)	NR	NR	NR	NR
Disease activity								
		(n = 11)	(n = 31)	(n = 42)				
PASI [mean (SD)]		7.42 (7.45)	3.69 (6.79)	4.62 (7.05)	**0.037**	**0.030**	0.993	0.139
Dermatological activity (n; %)		3 (27.2%)	4 (12.9%)	7 (16.7%)	0.547	0.353	0.750	0.416
Rheumatic activity (n; %)		–	10 (32.3%)	10 (23.8%)	–	–	–	–
Metabolic parameters								
BMI [mean; kg/m²; (SD)]		28.72 (9.52)	27.73 (4.78)	27.99 (6.27)	0.785	> 0.999	> 0.999	> 0.999
BMI > 25 kg/m² (n; %)		8/11 (72.7%)	23/30 (76.7%)	31/41 (75.6%)	0.967	> 0.999	0.918	> 0.999
HOMA [mean (SD)]		3.22 (2.41)	3.93 (3.73)	3.74 (3.41)	0.953	> 0.999	> 0.999	> 0.999
HOMA > 3 (n; %)		4 (76.4%)	12/30 (40%)	16/41 (39%)	0.978	> 0.999	0.934	> 0.999
Metabolic syndrome (n; %)		4 (36.4%)	10 (32.3%)	14 (33.3%)	0.970	> 0.999	0.923	> 0.999
Abdominal circumference [mean; cm; (SD)]	Female	116 (35.36) (n = 2)	92.8 (12.47) (n = 11)	96.4 (17.60) (n = 13)	0.539	0.800	> 0.999	> 0.999
	Male	99.3 (20.93) (n = 8)	103.2 (12.10) (n = 11)	101.5 (15.99) (n = 19)	0.799	> 0.999	> 0.999	> 0.999
High Abdominal circumference (n; %)		6/10 (60%)	19/22 (86.4%)	25/32 (78.1%)	0.247	0.165	0.501	0.410
CRP [mean; mg/L; (SD)]		8.18 (19.95)	8.78 (14.57)	8.62 (15.92)	0.329	0.408	> 0.999	0.773
High CRP (n; %)		4 (36.4%)	19 (61.3%)	23/41 (56.1%)	0.358	0.180	0.658	0.317

NR, not relevant. Significant values are bold.

### 3.2 Cytokine profiles of psoriatic disease

Since PsD is a systemic disorder, analysis of all patients taken together was first performed before deeper analysis depending on clinical phenotype.

#### 3.2.1 Comparison of PsD patients with healthy subjects

**Figure 1:**
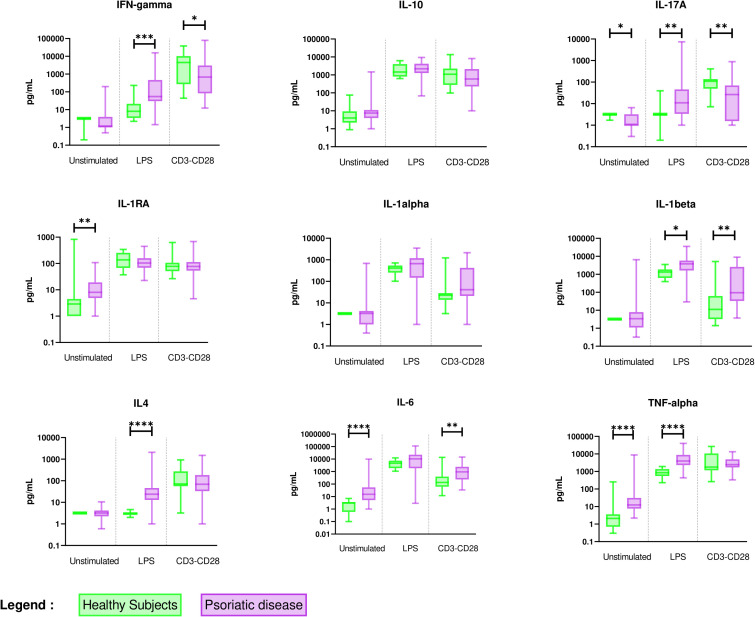
Overall comparison of *ex vivo* cytokine production in PBMC supernatants between healthy subjects (n = 11) and PsD patients (n = 48). PBMCs from healthy subjects (n = 11) and PsD patients (n= 48) were cultured at 1x10^6^ cells/ml for 24 h at 37°C, 5% CO_2_ with and without stimulation of monocytes by *E. coli* LPS (1 µg/mL) or T lymphocytes by anti-CD3/CD28 mAb (2.5 × 10^4^ beads/10^6^ cells/ml). Supernatants were collected and cytokine levels were assayed by Luminex Multiplex ELISA. Results are presented using median values with minimum to maximum box-plot. Difference between the groups using non-parametric Mann-Whitney U test is presented in the top of the figure for each condition. *p < 0.05; **p < 0.01; ***p < 0.001; ****p < 0.0001.

In comparison with healthy subjects, unstimulated PBMC of PsD patients exhibited a monocyte-predominant pro-inflammatory pattern with higher spontaneous release of IL-6 and TNF-α and higher release of IL-1RA. Stimulation of the monocytes by LPS induced higher levels of IL-1β and higher secondary activation of Th1 (IFN-γ), Th2 (IL-4), and Th17 (IL-17A) cytokines. In addition, the activation of T lymphocytes by anti CD3/CD28 mAb indirectly stimulated monocyte production of IL-1β at a higher level than in healthy controls. IL-6 higher production could result from either directly stimulated T-cells or indirectly activated monocytes. Surprisingly, these stimulated lymphocytes displayed lower levels of IFN-γ and IL-17A than healthy controls (**Figure 1** and **Supplementary Table 1**).

Those results suggested a pronounced inflammatory state of monocytes able to secondarily activate different T cell subtypes in PsD patients when compared to healthy controls, whereas direct T cell activation in PsD patients was reduced.

#### 3.2.2 Cytokine levels according to biological and clinical parameters of MetS

Differences in cytokine secretion between PsD patients with high circulating CRP (> 3 mg/L), BMI above 25 kg/m², HOMA-score above 3, presence of MetS, or elevated abdominal circumference and PsD patients without these features were analysed (**Table 2**).

**Table 2:** Comparison of *ex vivo* cytokine production depending on MetS and related parameters in PsD patients (median; pg/mL).

Cytokine	Stimulation	High CRP (> 3 mg/L)			BMI > 25 kg/m²			HOMA score > 3			MetS			High abdominal circumference		
		+	-	*p*	+	-	*p*	+	-	*p*	+	-	*p*	+	-	*p*
IFN-γ	None	1	1	ns	1	3	ns	1	1	ns	1	1	ns	1	1	ns
	LPS	55.50	51.70	ns	55.5	45.55	ns	53.90	58.00	ns	36	132.5	**0.014**	55	58	ns
	CD3-CD28	178.8	2209	ns	390.5	1249	ns	1847	190.6	ns	947.8	406.3	ns	789	565	ns
IL-10	None	8.30	5.75	ns	7.60	6.65	ns	7.3	7.6	ns	5.6	7.6	ns	7	8	ns
	LPS	2212	2500	ns	2364	1758	ns	2495	2076	ns	1569	2791	ns	2860	2765	ns
	CD3-CD28	809.2	589	ns	848.5	483	ns	692.2	809.2	ns	692.2	706.2	ns	809	501	ns
IL-17A	None	1.0	1.20	ns	1.0	1.0	ns	1.0	1.0	ns	1.0	1.0	ns	1	1	ns
	LPS	19.80	9.30	**0.020**	10.30	19.75	ns	9.55	20.40	ns	10	19	ns	10	20	ns
	CD3-CD28	21.30	34.80	ns	23.70	28.70	ns	36.25	17.00	ns	36	20	ns	35	70	ns
IL-1RA	None	9.0	5.45	ns	8.25	7.85	ns	4.65	7.85	ns	7	8	ns	7	8	ns
	LPS	112.2	107.5	ns	104.4	102.3	ns	124.6	97.20	ns	125	101	ns	137	111	ns
	CD3-CD28	113.0	62.70	**< 0.001**	98.70	72.90	ns	79.65	81.70	ns	77	88	ns	99	77	ns
IL-1α	None	3.3	3.2	ns	3.40	1.30	ns	3.50	2.60	ns	3	3	ns	3	3	ns
	LPS	521.5	665	ns	521.5	770.2	ns	721.4	513.7	ns	510	710	ns	598	886	ns
	CD3-CD28	128	32.50	**0.031**	58.90	49.75	ns	47.10	59.60	ns	33	59	ns	42	40	ns
IL-1β	None	4.10	2.45	ns	3.6	3.4	ns	2.75	4.2	ns	3	4	ns	3	5	ns
	LPS	3746	3363	ns	2723	5592	**0.017**	3363	2971	ns	3059	3641	ns	3190	5369	ns
	CD3-CD28	156.9	77.85	ns	90.90	119.3	ns	83.45	149.6	ns	125	110	ns	80	139	ns
IL-4	None	3.40	2.850	ns	3.2	3.0	ns	3.15	3.40	ns	3	3	ns	3	4	ns
	LPS	38.40	19.40	**< 0.001**	25.10	24.0	ns	21.10	26.50	ns	28	25	ns	23	27	ns
	CD3-CD28	66.0	143.1	ns	53.70	144.0	ns	86.95	66.0	ns	160	52	ns	90	283	ns
IL-6	None	22.80	11.70	ns	18.60	7.85	ns	15.75	17.70	ns	12	16	ns	13	11	ns
	LPS	6144	16420	ns	12084	10428	ns	17133	10000	ns	9939	12248	ns	13693	16098	ns
	CD3-CD28	1911	482.7	**0.030**	1409	569	ns	963.5	1059	ns	1473	902	ns	870	567	ns
TNF-α	None	12.40	11.45	ns	12.50	10.10	ns	12.40	11.20	ns	11	12	ns	12	11	ns
	LPS	6875	3389	ns	5878	3185	ns	5984	3945	ns	3135	4597	ns	3576	3945	ns
	CD3-CD28	2196	3150	ns	2374	2336	ns	3026	2125	ns	2285	2834	ns	2546	3926	ns

ns, not significant. Significant values are in bold.

In patients with high CRP, LPS-induced monocyte production of IL-17A and IL-4 was higher than in patients with low CRP, while direct stimulation of T cells had an indirectly stronger monocyte-stimulating effect with higher secretion of IL-1α and IL-6. PBMC from patients with high BMI secreted lower levels of IL-1β after LPS stimulation than those from normal BMI patients. When considering patients with MetS, stimulated monocytes were less prone to stimulate Th1 cells (less IFN-γ release) than those without MetS. Regarding these parameters, no difference was observed regarding HOMA-score or abdominal circumference.

#### 3.2.3 Cytokine levels according to cutaneous or rheumatic activity in PsD patients

**Figure 2:**
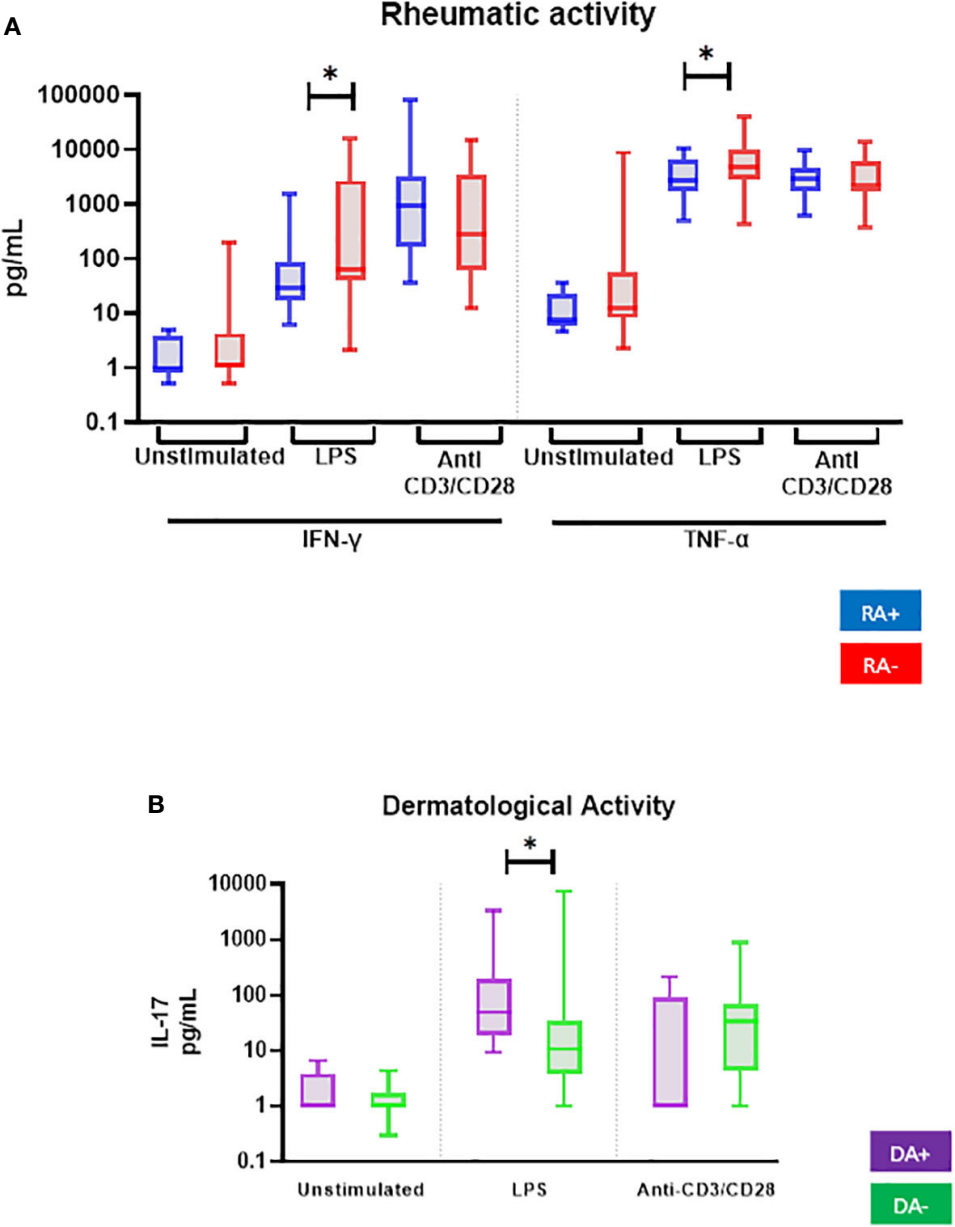
Comparison of *ex vivo* cytokine production of all PsD patients (n = 48) according to **(A)** their rheumatic (RA) and **(B)** dermatological (DA) disease activity. PBMCs from PsD patients (n = 48) were cultured at 1x10^6^ cells/ml for 24 h at 37°C, 5% CO_2_ with and without stimulation of monocytes by *E. coli* LPS (1 µg/mL) or T lymphocytes by anti-CD3/CD28 mAb (2.5 × 10^4^ beads/10^6^ cells/ml). Supernatants were collected and cytokine levels were assayed by Luminex Multiplex ELISA. Results are presented using median values with minimum to maximum box-plot. Difference between the groups using non-parametric Mann-Whitney U test is presented in the top of the figure for each condition. *p < 0.05. RA, Rheumatic activity (n RA+ = 10); DA, Dermatological activity (n DA+ = 7).

PBMC from patients with rheumatic activity (10 of 42) showed lower IFN-γ and TNF-α production after LPS stimulation (**Figure 2A** and **Table 3**). LPS-stimulated PBMC from patients with dermatological activity (7 of 42 PsD patients) displayed higher IL-17A production (**Figure 2B**).

**Table 3:** Comparison of *ex vivo* cytokine production depending on disease activity (dermatological or rheumatic) in PsO patients, PsA patients, and PsD (median; pg/mL).

Cytokine	Stimulation	PsO		PsA				PsD						Dermatological Activity PsO	Dermatological Activity PsA	Dermatological Activity PsD	Rheumatic Activity PsA	Rheumatic Activity PsD	Overall Activity PsD
		DA+	DA-	DA+	DA-	RA+	RA-	DA+	DA-	RA+	RA-	OA+	OA-						
IFN-γ	None	4.700	1.050	1.000	1.200	1.000	1.100	1.000	1.100	1.000	1.150	1.000	1.150	ns	ns	ns	ns	ns	ns
	LPS	143.6	47.20	2244	50.30	30.00	135.5	143.6	50.30	28.60	64.90	45.25	56.75	ns	ns	ns	**0.029**	**0.011**	ns
	CD3-CD28	390.5	2137	96.20	422.1	789.2	158.7	114.8	789.2	947.8	290.6	605.7	377.6	ns	ns	ns	ns	ns	ns
IL-10	None	34.60	4.700	4.750	7.600	7.600	7.300	7.000	7.600	7.500	7.300	7.500	6.900	ns	ns	ns	ns	ns	ns
	LPS	3064	2812	1263	1935	1935	1716	2076	2235	2150	2223	2220	2223	ns	ns	ns	ns	ns	ns
	CD3-CD28	3049	507.0	1515	80.2	817.9	916.9	1800	574.8	813.6	589.0	987.7	533.8	ns	ns	ns	ns	ns	ns
IL-17A	None	2.900	1.200	1.000	1.000	1.700	1.000	1.000	1.000	1.350	1.000	1.350	1.000	ns	ns	ns	ns	ns	ns
	LPS	20.40	5.250	123.0	18.90	11.30	20.10	49.80	10.70	10.45	19.10	19.25	14.80	ns	ns	**0.032**	ns	ns	ns
	CD3-CD28	25.40	35.55	1.000	21.30	55.60	8.650	1.000	34.00	50.95	18.20	35.85	18.20	ns	ns	ns	ns	ns	ns
IL-1RA	None	51.30	8.400	5.450	7.650	6.200	7.300	7.300	8.050	7.100	8.100	7.650	8.100	ns	ns	ns	ns	ns	ns
	LPS	104.4	176.0	86.15	97.20	148.1	65.65	99.00	112.2	153.4	98.10	128	99.90	ns	ns	ns	**0.017**	ns	ns
	CD3-CD28	109.4	65.60	107.0	81.70	82.90	80.25	109.4	78.80	84.40	80.25	99.65	77.60	ns	ns	ns	ns	ns	ns
IL-1α	None	6.300	2.900	1.500	3.300	3.300	2.800	2.000	3.300	3.350	2.800	3.350	2.800	ns	ns	ns	ns	ns	ns
	LPS	886.4	1570	58.15	513.7	621.2	417.0	94.20	642.8	654.2	556.1	609.8	578.3	ns	ns	ns	ns	ns	ns
	CD3-CD28	258.1	23.60	477.7	79.40	43.40	124.7	258.1	42.30	42.85	59.25	69.15	37.95	ns	ns	ns	ns	ns	ns
IL-1β	None	13.80	1.800	6.550	2.800	4.200	3.200	7.600	2.700	3.450	3.850	4.650	2.450	ns	ns	ns	ns	ns	ns
	LPS	5329	6820	1886	2929	2971	2496	3536	3190	3444	3363	3253	3468	ns	ns	ns	ns	ns	ns
	CD3-CD28	1464	41.55	2670	128.7	86.70	242.5	1464	86.70	74.15	170.9	95.20	144.3	ns	ns	ns	ns	ns	ns
IL-4	None	4.900	2.750	4.100	3.200	3.400	3.100	4.900	3.000	3.600	3.000	3.600	3.000	ns	ns	ns	ns	ns	ns
	LPS	25.10	24.25	427.1	25.10	21.50	26.95	98.20	25.10	23.30	25.80	25.10	25.80	ns	ns	ns	ns	ns	ns
	CD3-CD28	51.10	182.0	101.8	53.70	154.7	48.90	51.10	90.30	144.0	52.40	101.1	72.00	ns	ns	ns	ns	ns	ns
IL-6	None	182.3	8.550	11.90	13.00	7.100	15.35	87.70	12.90	14.95	15.35	23.40	11.70	**0.049**	ns	ns	ns	ns	ns
	LPS	31654	20450	649.0	10000	13639	8398	2707	12084	15801	10428	15191	10428	ns	ns	ns	ns	ns	ns
	CD3-CD28	2488	563.0	2808	1059	1409	1058	2488	1016	1647	943.2	1899	772.6	ns	ns	ns	ns	ns	ns
TNF-α	None	63.60	10.40	13.05	11.70	7.600	12.45	25.90	11.20	7.850	12.45	13.35	11.45	**0.049**	ns	ns	ns	ns	ns
	LPS	6453	5279	4580	4005	2391	4697	5878	4005	2633	4697	3614	4184	ns	ns	ns	**0.026**	**0.048**	ns
	CD3-CD28	1672	2180	4805	2546	2930	2371	3238	2374	2855	2285	3084	2161	ns	ns	ns	ns	ns	ns

ns, not significant. Significant values are in bold. DA, dermatological activity; RA, rheumatic activity; OA, overall activity.

#### 3.2.4 Comparison between PsO and PsA patients

**Figure 3:**
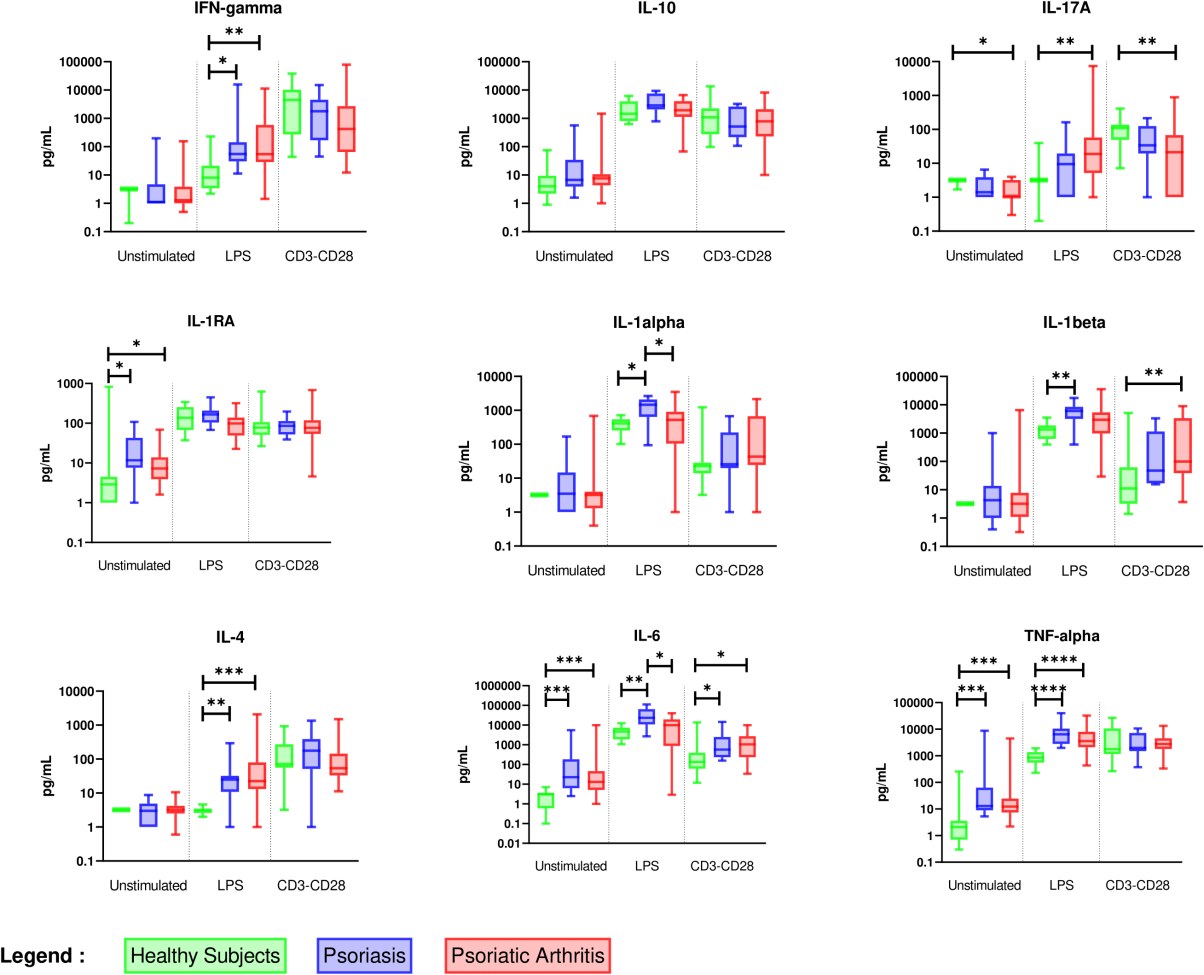
Overall comparison of *ex vivo* cytokine production between healthy subjects (n = 11), PsO (n = 11), and PsA (n = 37) patients. PBMCs from healthy subjects (n = 11), PsO (n = 11), and PsA (n = 37) patients were cultured at 1x10^6^ cells/ml for 24 h at 37°C, 5% CO_2_ with and without stimulation of monocytes by *E. coli* LPS (1 µg/mL) or T lymphocytes by anti-CD3/CD28 mAb (2.5 × 10^4^ beads/10^6^ cells/ml). Supernatants were collected and cytokine levels were assayed by Luminex Multiplex ELISA. Results are presented using median values with minimum to maximum box-plot. Difference between the groups using non- parametric Kruskal-Wallis test with Dunn’s multiple comparison test is presented at the top of the figure for each condition. *p < 0.05; **p < 0.01; ***p < 0.001; ****p < 0.0001.

PsO and PsA patients (with all rheumatologic phenotypes encompassed) were compared (**Figure 3** and **Supplementary Table 2**). Unstimulated PBMC from both PsO and PsA displayed higher levels of spontaneous release of IL-6, TNF-α, and IL-1RA without difference between the two. Stimulation of monocytes by LPS induced higher activation of Th1 and Th2 cells, while T cell activation induced higher production of IL-6 in the two groups. A spontaneous inflammatory state (IL-6, TNF-α and IL-1RA) with monocyte-mediated activation of Th1 (IFN-γ, IL-6) cells is a common feature of PsO and PsA patients.

However, only PsO patients displayed an increased production of IL-1α, IL-1β, and IL-6 by stimulated monocytes, indicating a monocyte-predominant cytokine signature of PsO.

In PsA patients, stimulated monocytes activated Th17 cells, while T lymphocyte-stimulation reduced IL-17A but enhanced IL-1β production when compared to healthy controls. This finding suggests that Th17 cells need interaction with stimulated monocytes to produce IL-17A, while their own stimulation activated only monocytes in PsA.

### 3.3 Cytokine profiles in psoriatic arthritis

#### 3.3.1 Cytokine profile of each clinical phenotype of PsA

**Figure 4:**
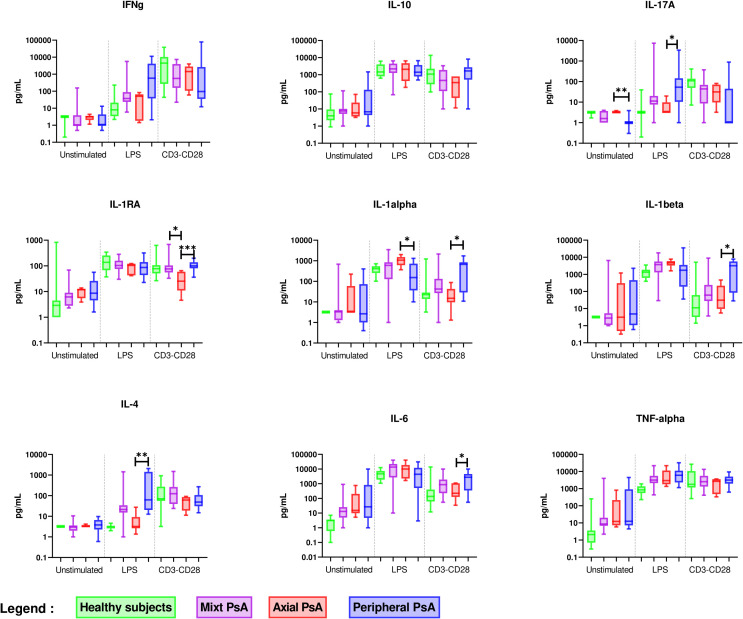
Comparison of *ex vivo* cytokine production between axial (n = 6), peripheral (n = 14) and mixed PsA (n = 17). PBMCs from axial (n = 6), peripheral (n = 14) and mixed PsA (n = 17) patients were cultured at 1x10^6^ cells/ml for 24 h at 37°C, 5% CO_2_ with and without stimulation of monocytes by *E. coli* LPS (1 µg/mL) or T lymphocytes by anti-CD3/CD28 mAb (2.5 × 10^4^ beads/10^6^ cells/ml). Supernatants were collected and cytokine levels were assayed by Luminex Multiplex ELISA. Results are presented using median values with minimum to maximum box-plot. Difference between the groups using non parametric Kruskal-Wallis test with Dunn’s multiple comparison test is presented in the top of the figure for each condition. *p < 0.05; **p < 0.01; ***p < 0.001.

Considering patients with PsA, we further identified the specific signature of each clinical phenotype that would reveal distinctive underlying pathways (**Figure 4** and **Supplementary Table 3**). Peripheral PsA exhibited a cytokine profile distinct from axial PsA, characterized by Th17 and Th2 activation by activated monocytes and higher activation of monocytes by stimulated lymphocytes. Therefore, a specific peripheral PsA cytokine signature is characterised by a stronger monocyte activation with higher IL-6, IL-1α, and IL-1β levels after T lymphocyte stimulation, as well as greater activation of Th17 and Th2 cells after monocyte stimulation.

The features distinguishing axial PsA from peripheral PsA were higher production of IL-1α by stimulated monocytes, which is part of the PsO cytokine signature, and a lower production of IL-4 after monocyte stimulation. The singularity of an axial PsA cytokine profile is the absence of Th2 cell activation by stimulated monocytes.

#### 3.3.2 *Ex vivo* cytokine profiles according to disease activity

As regards rheumatic activity in the 31 PsA patients, a Th1 and monocytic-predominant signature appeared. Higher production of IL-1RA after LPS stimulation was found, as was the case for IFN-γ and TNF-α as previously described in the PsD patients. No difference was observed according to the dermatological activity of PsA (**Table 3**).

#### 3.3.3 Impact of DMARD initiation on cytokine profiles in psoriatic arthritis

**Figure 5:**
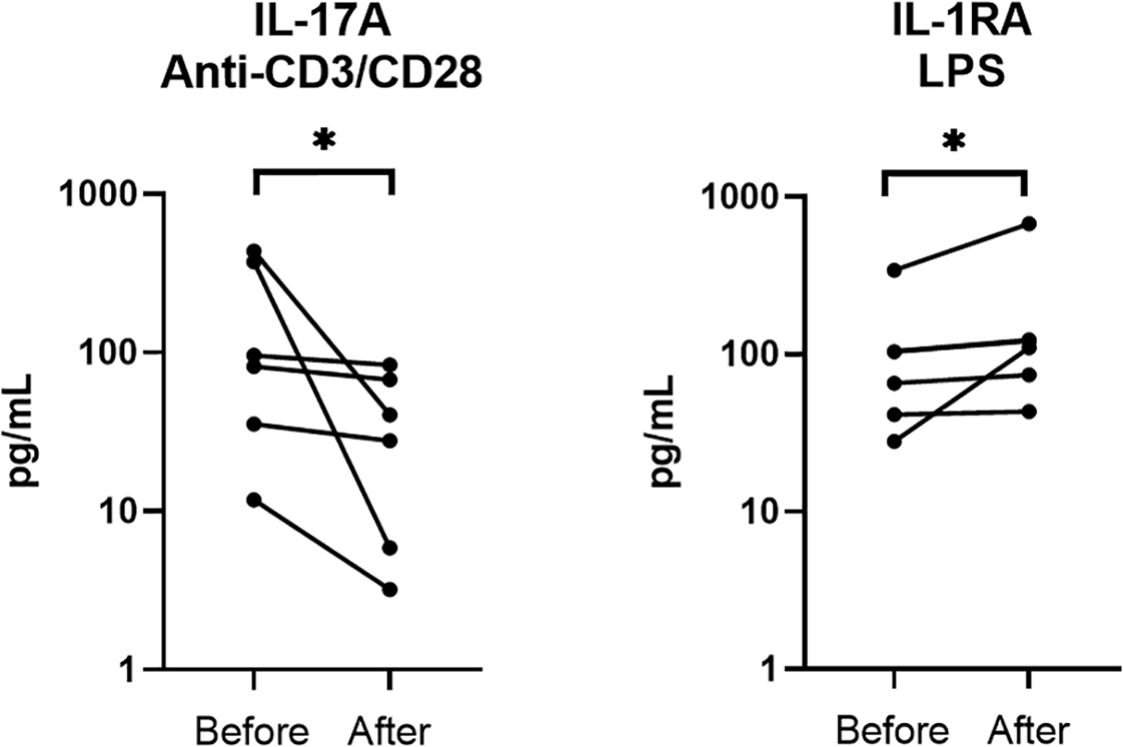
Comparison of *ex vivo* cytokine production in PsA patients before and after DMARD initiation (n = 6). PBMCs from 6 PsA patients were cultured at 1x10^6^ cells/ml for 24 h at 37°C, 5% CO_2_ with and without stimulation of monocytes by *E. coli* LPS (1 µg/mL) or T lymphocytes by anti-CD3/CD28 mAb (2.5 × 10^4^ beads/10^6^ cells/ml). Supernatants were collected and cytokine levels were assayed by Luminex Multiplex ELISA. Difference between the groups using non-parametric Wilcoxon matched-pairs test is presented in the top of the figure for each condition. *p < 0.05. Each line represents a patient.

Results of *ex vivo* cytokine production by PBMC before and after DMARD initiation were available for 6 patients (**Figure 5**). After treatment, lower production of IL-17A after anti-CD3/CD28 mAb stimulation and higher production of IL-1RA after LPS stimulation were observed, suggesting that the PsA treatment reduced Th17 pro-inflammatory state and reinforced the control of inflammation by monocytes through IL-1RA release.

## 4 Discussion

This exploratory study analysed the *ex vivo* cytokine production by PBMC stimulated with either LPS or anti-CD3/CD28 mAb in clinically well-described populations of PsA and PsO patients (26, 27). PBMCs have the advantage of being easily accessible to perform cytokine measurements after stimulation, although in inflammatory arthritis many other cell types produce cytokines, including fibroblasts, synoviocytes, immune infiltrated cells and osteoblasts. These cells can contribute to the cytokine network of the disease, particularly inside the synovium, and may have different cytokine production patterns (28–31). However, isolating these infiltrated cells is not easy since it requires invasive techniques, making it not suitable for a daily analysis, by contrast to PBMC *ex vivo* cytokine production (32).

In our study, LPS and anti-CD3/CD28 mAb stimulation of PBMCs were used to stimulate monocytes and T-cells, respectively (23, 24). LPS is a TLR4 agonist traditionally considered as a monocyte activator since TLR4 expression is mainly expressed in myeloid cells in humans although minor expression level of TLR4 has been also described in some lymphoid cell subsets (33). Concerning the activation of T cells following LPS stimulation, the literature is contradictory. Indeed some authors found a direct activation of T-cell by LPS through TLR4 (34) meanwhile some authors hypothesize that it is the result of cell-cell interactions with monocytes. Recent single cell analyses of LPS or anti-CD3/CD28 stimulated-PBMC confirm that LPS only activate monocytes (35). Considering this recent literature, we analysed our results considering that LPS activates monocytes and not T-cells, even if a marginal activation of T-cells could not be ruled out.

Several studies have attempted to determine specific cytokine profiles of PsD. Increased IL-17A production by PBMC after 4 days of anti-CD3/CD28 mAb stimulation has been described in PsO and PsA patients (36). Another study reported high IL-17A in supernatants of CD4^+^ T cells after 6 days of culture with anti-CD3/CD28 mAb stimulation in very early PsA (37), while we report lower IL-17A levels with similar conditions of stimulation. In our study, 24h of stimulation were used to limit secondary or tertiary production of cytokines that can modify the profile following longer stimulation. Another explanation for this result may be the need for interactions with other cell types and not only T cell stimulation to induce higher IL-17A levels early in PsD. In accordance with Leijten et al. (38), higher IL-1RA levels were observed in unstimulated conditions compared to healthy controls, which reflects an attempt by the immune system to alleviate systemic inflammation. Interestingly, while LPS stimulation induced increased production of all the cytokines studied in PsD patients, no increase of IL-4, IL-17, and IFN-γ was observed in healthy controls. All in all, this suggests a particular ability of circulating T cells to be activated *via* monocytes in PsD.

Patients suffering from PsD are known to present MetS features, a clinical entity with complex and multiple impacts on the immune system, as reviewed by Andersen et al. (39). There is no literature on *ex vivo* PBMC-derived cytokine profiles comparing patients according to their BMI status. A recent study showed an exhaustion of T cells inside adipose tissue of obese patients, which is concordant with our findings (40). As reported by Diehl et al., IL-6 exerts an inhibitory effect on Th1 differentiation, thereby lowering IFN-γ production (41). Therefore, since LPS stimulates production of IL-6 by PBMC, we can hypothesize that stimulated monocytes polarize toward a pro-inflammatory phenotype with increased production of IL-6, which hampers IFN-γ production by Th1 cells, especially as IL-6 levels did not differ between patients with and without MetS.

Comparison of the cytokine profiles of the different clinical phenotypes of PsD patients demonstrated features distinctive to each subtype. In PsO patients, our results confirm previous studies highlighting the pivotal role of monocytes and their cytokines (IL-1α, IL-1β, and IL-6) (42–44). Likewise, clinical improvement of PsO with biological therapies has been associated with decreased monocyte activity (45). Since IL-1β induces IL-6 secretion by monocytes (46), we hypothesize that a cascade of cell activation might contribute to IL-6 production. Nevertheless, IL-6 implication in PsO pathogenesis remains unclear, and its inhibition in clinical practice is not efficient (47). Intriguingly, IL-17A is not present in PsO cytokine signature, whereas IL-17A is one of the main cytokines that drives cutaneous inflammation in psoriasis (48). However, activated monocytes may be one of the missing links. In fact, IL-1β enhances the differentiation and expansion of Th17 cells (49). Moreover, the IL-1β/IL-1R pathway regulates dermal IL-17A-producing cells and the stimulation of keratinocytes in PsO (50). Considering the absence of IL-17A expression by PBMCs from PsO patients, we hypothesize that IL-17A might be produced essentially by tissue-resident cells such as type 3 innate lymphoid cells (ILC3) for instance (51).

The specific cytokine profile of peripheral PsA raises the interesting concept of crosstalk between immune cells subtypes as a crucial element of disease pathophysiology. In 1997, Chizzolini et al. demonstrated that activated cells could induce the production of cytokines by monocytes through cell-cell contact (52). In 2009, Evans et al. demonstrated that LPS-activated monocytes induce Th17 responses when co-cultured with CD4^+^ T cells, thereby highlighting the importance of monocyte/T cell interaction in the shaping of inflammatory T cell responses (53). These interactions seem to be the key to the vicious circle of inflammation found in peripheral but not in axial PsA. It is also well-known that peripheral PsA patients present a more inflammatory phenotype, reflected by higher levels of erythrocyte sedimentation rate or CRP than axial PsA patients (54). We can parallel a study focusing on innate lymphoid cells (ILCs) in different rheumatic diseases with either a synovitis-predominant pattern or not. Differences in ILC populations were found between these two groups, but not between the different rheumatic diseases, implying that the clinical profile may be more important than the disease itself to distinguish particular immune patterns (55). The importance of T cell activation in PsA has also been highlighted in a study reporting a therapeutic strategy based on targeted treatments in which patients with an activated Th17 predominant pattern were treated by an IL-17A inhibitor, while patients harbouring an activated Th1 predominant pattern received an anti-p40 treatment. This study showed better treatment efficacy, highlighting the importance of T cells in the pathophysiology of peripheral PsA (56).

Focusing on disease activity, the spontaneous production of IL-6 and TNF-α was increased in patients with dermatological activity, as previously reported for TNF-α, but not for IL-6 (57). In accordance with our results showing an increase of IL-17A after LPS stimulation in PsD patients with dermatological activity, it has been reported that IL-17^+^ T cells were increased in active psoriasis (58, 59) and that elevated concentrations of IL-17A in serum were correlated with the dermatological activity (60). In PsA, in agreement with our results, serum IL-1RA was associated with joint severity and correlated with tender and swollen joints (12). Lower levels of IFN-γ associated with rheumatic disease activity was to be expected, given the accumulating evidence of a “yin and yang” effect of IFN-γ on the inflammatory process (61), particularly with negative regulation of IL-17 expression by Th17 cells (62).

After DMARD initiation, we observed decreased IL-17A production and increased IL-1RA production, in accordance with the decreased inflammatory process. Previous studies reported not only decreased serum IL-17A levels (63), but also decreased TNF-α after TNF-α inhibitor treatment, which was not observed in our *ex vivo* analysis (64, 65). It is noteworthy that the approaches were different, and that, to our knowledge, no *ex vivo* PBMC stimulation study has focused on this issue.

One of the limits of this study is the inclusion of subjects who, for the most of part, already benefited from DMARDs therapies, which have an impact on cytokines production (66). However, this feature equally concerns both PsO and PsA patients, thereby limiting the bias. The number of patients included is also limited but comparable to other studies in the field (10, 11, 13, 14, 16, 17). In any event, our results should be viewed with all the necessary precautions. While the ex *vivo* approach is dynamic and easy to implement to study the contribution of circulating cells to the diseases, it excludes the contribution of resident cells. Another limit of this global approach is that the underlying mechanisms of the complex interactions between immune cells and their subsequent cytokine productions were not fully elucidated.

**Figure 6:**
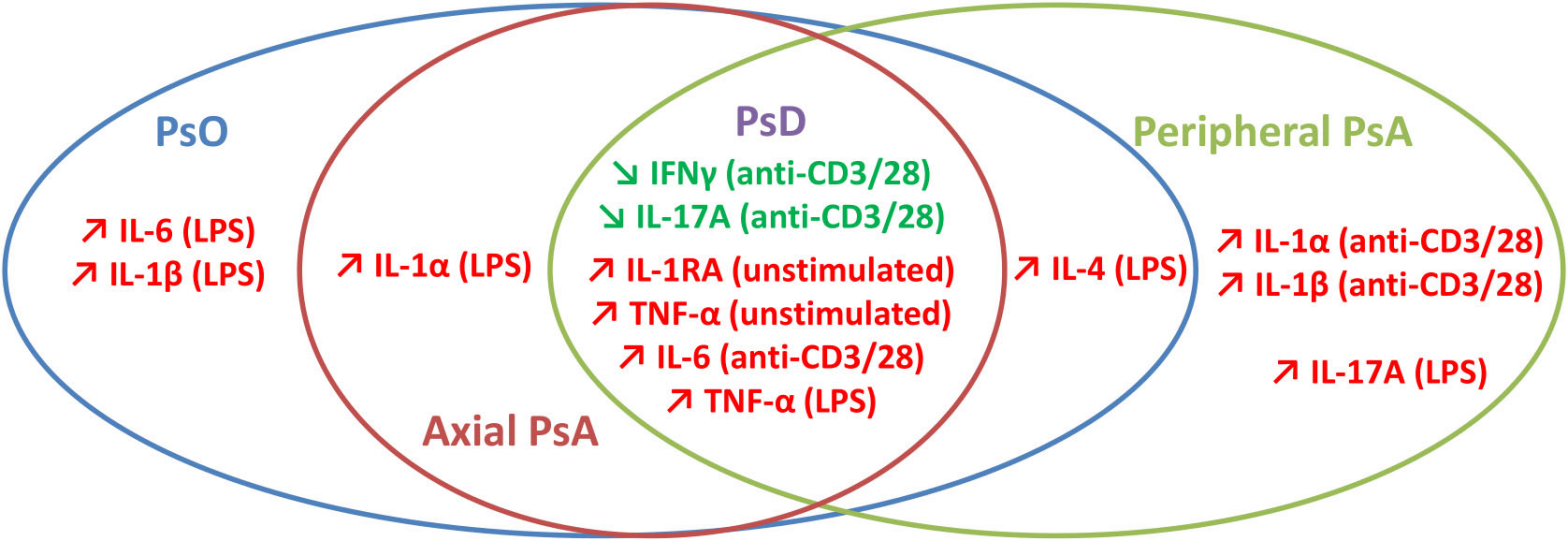
Synthetic view of discriminative *ex vivo* cytokine profiles in psoriatic disease.

Taken together, our results lead us to propose a core cytokine profile for all PsD patients and specific cytokine patterns distinguishing the different PsD sub-phenotypes, as summarized in **Figure 6**. The perspective of characterizing distinct particular immune profiles might be of great interest to tailor patient treatment with a potential clinical impact on the effectiveness of biologic DMARDs (56).

In conclusion, PsO and PsA share not only the common cytokine patterns common to PsD, but a number of specific cytokine patterns, as well. PsO exhibits a monocyte-predominant cytokine signature, whereas peripheral PsA signature is characterized by strong monocyte activation by stimulated T cells and Th17 cell activation by stimulated monocytes. Our study highlights the crucial role of immune cell interaction potentially involving soluble mediators and/or cell-cell contact with different patterns of interaction depending on clinical phenotype. Further studies should be performed to increase knowledge of the immunological mechanisms that underlie every sub-phenotype of PsD.

## Data availability statement

The raw data supporting the conclusions of this article will be made available by the authors, without undue reservation.

## Ethics statement

The studies involving human participants were reviewed and approved by Poitiers University Hospital. Written informed consent for participation was not required for this study in accordance with the national legislation and the institutional requirements.

## Author contributions

AD, TD, and PV performed the experiments. CS, EG, and PV acquired data. GL, AD, TD, PV, CS, FM, J-CL, and EG analysed data. GL performed statistical analysis. GL wrote first draft of the manuscript. GL, EG, FM, J-CL, and J-FJ corrected manuscript. All authors contributed to the article and approved the submitted version.

## Funding

This research was supported by Poitiers University Hospital and Poitiers University.

## Acknowledgments

Pr. Stéphanie RAGOT for her statistical advices and validation. M. Jeffrey Arsham for language editing of the manuscript. The authors thank the European Union for an equipment grant program for research laboratories and platforms.

## Conflict of interest

The authors declare that the research was conducted in the absence of any commercial or financial relationships that could be construed as a potential conflict of interest.

## Publisher’s note

All claims expressed in this article are solely those of the authors and do not necessarily represent those of their affiliated organizations, or those of the publisher, the editors and the reviewers. Any product that may be evaluated in this article, or claim that may be made by its manufacturer, is not guaranteed or endorsed by the publisher.
