# Supplementary material for: Ex vivo cytokine production in psoriatic disease: Towards specific signatures in cutaneous psoriasis and peripheral psoriatic arthritis
Source: Front Immunol. 2022 Nov 8;13:993363. doi: 10.3389/fimmu.2022.993363 (PMC9678922; doi:10.3389/fimmu.2022.993363)
Supplement: Supplementary file 1 [file Table_1.docx]

Supplementary Table 1 : Overall comparison of *ex vivo* cytokine production between healthy subjects (n = 11) and PsD (n = 48)

| Cytokine | Stimulation | Healthy  (median)  pg/mL | PsD  (median)  pg/mL | p |
| --- | --- | --- | --- | --- |
| IFN-γ | None | 3,2 | 1.20 | ns |
|  | LPS | 8,1 | 54.70 | **< 0.001** |
|  | CD3-CD28 | 4475 | 676.9 | **0.030** |
| IL-10 | None | 4 | 7.50 | ns |
|  | LPS | 1453 | 2212 | ns |
|  | CD3-CD28 | 1094 | 589.0 | ns |
| IL-17A | None | 3,2 | 1.0 | **0.013** |
|  | LPS | 3,2 | 11.0 | **0.005** |
|  | CD3-CD28 | 109,8 | 26.57 | **0.003** |
| IL-1RA | None | 2,9 | 8.050 | **0.003** |
|  | LPS | 137,5 | 104.5 | ns |
|  | CD3-CD28 | 78,2 | 77.60 | ns |
| IL-1α | None | 3,2 | 3.25 | ns |
|  | LPS | 410 | 665.0 | ns |
|  | CD3-CD28 | 22,8 | 41.10 | ns |
| IL-1β | None | 3,2 | 3.4 | ns |
|  | LPS | 1347 | 3804 | **0.013** |
|  | CD3-CD28 | 11,2 | 95.20 | **0.005** |
| IL-4 | None | 3,2 | 3.2 | ns |
|  | LPS | 3,2 | 23.85 | **< 0.0001** |
|  | CD3-CD28 | 71,1 | 69.28 | ns |
| IL-6 | None | 3,2 | 15.35 | **< 0.0001** |
|  | LPS | 4691 | 10428 | ns |
|  | CD3-CD28 | 135,1 | 943.2 | **0.002** |
| TNF-α | None | 2,1 | 12.40 | **< 0.0001** |
|  | LPS | 856,5 | 3975 | **< 0.0001** |
|  | CD3-CD28 | 1789 | 2460 | ns |

Supplementary Table 2 : Overall comparison of *ex vivo* cytokine production between healthy subjects (n = 11), PsO patients (n = 11), and PsA patients (n = 37)

| Cytokine | Stimulation | Healthy  (median)  pg/mL | PsO  (median)  pg/mL | PsA  (median)  pg/mL | *p* | H vs PsO  *p* | H vs PsA  *p* | PsO vs PsA  *p* |
| --- | --- | --- | --- | --- | --- | --- | --- | --- |
| IFN-γ | None | 3,2 | 1,1 | 1.3 | ns | ns | ns | ns |
|  | LPS | 8,1 | 55,5 | 54,7 | **0.002** | **0.014** | **0.003** | ns |
|  | CD3-CD28 | 4475 | 1791 | 422.1 | **0.046** | ns | *ns* | ns |
| IL-10 | None | 4 | 6,7 | 7,6 | ns | ns | ns | ns |
|  | LPS | 1453 | 2860 | 1929 | ns | ns | ns | ns |
|  | CD3-CD28 | 1094 | 512,9 | 791.7 | ns | ns | ns | ns |
| IL-17A | None | 3,2 | 1,4 | 1 | **0.043** | ns | **0.036** | ns |
|  | LPS | 3,2 | 9,5 | 18.90 | **0.008** | ns | **0.008** | ns |
|  | CD3-CD28 | 109,8 | 34 | 21.3 | **0.006** | ns | **0.006** | ns |
| IL-1RA | None | 2,9 | 11,7 | 7,3 | **0.011** | **0.013** | **0.034** | ns |
|  | LPS | 137,5 | 166,4 | 99.0 | ns | ns | ns | ns |
|  | CD3-CD28 | 78,2 | 85,9 | 76.80 | ns | ns | ns | ns |
| IL-1α | None | 3,2 | 3,5 | 3,2 | ns | ns | ns | ns |
|  | LPS | 410 | 1465 | 521.5 | **0.006** | **0.015** | ns | **0.020** |
|  | CD3-CD28 | 22,8 | 25,7 | 43.40 | **ns** | ns | **ns** | ns |
| IL-1β | None | 3,2 | 4,3 | 3,2 | ns | ns | ns | ns |
|  | LPS | 1347 | 6061 | 2971 | **0.004** | **0.002** | ns | **ns** |
|  | CD3-CD28 | 11,2 | 47,7 | 99.50 | **0.007** | ns | **0.006** | ns |
| IL-4 | None | 3,2 | 3 | 3,2 | ns | ns | ns | ns |
|  | LPS | 3,2 | 25,1 | 22.60 | **< 0.001** | **0.008** | **< 0.001** | ns |
|  | CD3-CD28 | 71,1 | 177,3 | 53,7 | Ns | ns | ns | ns |
| IL-6 | None | 3,2 | 22,8 | 13 | **0.0001** | **< 0.001** | **0.0001** | ns |
|  | LPS | 4691 | 23376 | 10000 | **0.004** | **0.005** | ns | **0.018** |
|  | CD3-CD28 | 135,1 | 566,7 | 1057 | **0.011** | **0.046** | **0.012** | ns |
| TNF-α | None | 2,1 | 13 | 12,4 | **0.0001** | **< 0.001** | **< 0.001** | ns |
|  | LPS | 856,5 | 6453 | 3576 | **< 0.0001** | **< 0.0001** | **< 0.0001** | ns |
|  | CD3-CD28 | 1789 | 1986 | 2780 | ns | ns | ns | ns |

Supplementary Table 3 : Comparison of *ex vivo* cytokine production between axial (n = 6), peripheral (n = 14), and mixed (n = 17) PsA patients

| Cytokine | Stimulation | Mixed  (median)  pg/mL | Peripheral  (median)  pg/mL | Axial  (median)  pg/mL | *p* | M vs P  *p* | P vs A  *p* | M vs A  *p* |
| --- | --- | --- | --- | --- | --- | --- | --- | --- |
| IFN-γ | None | 1.000 | 1.100 | 3.2 | ns | ns | ns | ns |
|  | LPS | 37.40 | 585.0 | 50.34 | **0.034** | **ns** | **ns** | ns |
|  | CD3-CD28 | 564.6 | 96.20 | 1431 | ns | ns | ns | ns |
| IL-10 | None | 7.600 | 6.900 | 5.905 | ns | ns | ns | ns |
|  | LPS | 2212 | 1401 | 2071 | ns | ns | ns | ns |
|  | CD3-CD28 | 464.8 | 1662 | 346.8 | ns | ns | **ns** | ns |
| IL-17A | None | 1.600 | 1.000 | 3.2 | **0.002** | **ns** | **0.002** | ns |
|  | LPS | 11.30 | 53.45 | 3.2 | **0.014** | ns | **0.014** | ns |
|  | CD3-CD28 | 44 | 1 | 31.57 | ns | ns | ns | ns |
| IL-1RA | None | 6.200 | 8.600 | 11.87 | ns | ns | ns | ns |
|  | LPS | 103.6 | 86.65 | 104.1 | ns | ns | ns | ns |
|  | CD3-CD28 | 75.90 | 100.6 | 25.33 | **0.002** | ns | **< 0.001** | **0.032** |
| IL-1α | None | 3.300 | 2.650 | 3.2 | ns | ns | ns | ns |
|  | LPS | 598.4 | 153.4 | 1077 | **0.029** | ns | **0.024** | ns |
|  | CD3-CD28 | 42.30 | 668.4 | 15.07 | **0.016** | ns | **0.013** | **ns** |
| IL-1β | None | 2.800 | 4.850 | 3.150 | Ns | ns | ns | ns |
|  | LPS | 3746 | 1756 | 4710 | Ns | ns | ns | ns |
|  | CD3-CD28 | 61.60 | 3202 | 31.21 | **0.016** | **ns** | **0.030** | ns |
| IL-4 | None | 3.00 | 3.800 | 3.2 | Ns | ns | ns | ns |
|  | LPS | 21.50 | 63.00 | 3.2 | **0.005** | **ns** | **0.004** | **ns** |
|  | CD3-CD28 | 125.5 | 48.90 | 61.68 | ns | ns | ns | ns |
| IL-6 | None | 12.90 | 26.80 | 15.13 | ns | ns | ns | ns |
|  | LPS | 13639 | 4402 | 10000 | ns | ns | ns | ns |
|  | CD3-CD28 | 870.2 | 2736 | 224.7 | **0.043** | ns | **0.046** | ns |
| TNF-α | None | 8.700 | 12.45 | 12.28 | ns | ns | ns | ns |
|  | LPS | 3179 | 6068 | 2980 | ns | ns | ns | ns |
|  | CD3-CD28 | 2546 | 3180 | 2664 | ns | ns | ns | ns |
